# When *Salmonella* Strikes the Brain: A Systematic Review of Non-Typhoidal *Salmonella* Infections with Central Nervous System Involvement in Immunosuppressed Patients

**DOI:** 10.3390/pathogens15010019

**Published:** 2025-12-23

**Authors:** Giulia Turicchi, Marco Bongiovanni

**Affiliations:** 1 Division of Infectious Diseases, Ente Ospedaliero Cantonale, 6900 Lugano, Switzerland; giulia.turicchi@eoc.ch; 2 Faculty of Biomedical Sciences, University of Southern Switzerland, 6900 Lugano, Switzerland

**Keywords:** *Salmonella*, meningitis, immune-suppression, HIV

## Abstract

Central nervous system (CNS) infections caused by *Salmonella* species (spp.) are exceptionally rare in adults but are associated with significant morbidity and mortality, particularly in immunocompromised individuals. Clinical presentation is often nonspecific, including fever, headache, or altered mental status, while imaging may demonstrate meningeal enhancement, abscesses, or cytotoxic lesions. We present a systematic review of non-typhoidal *Salmonella* spp. infections involving the CNS across various immunosuppressive contexts, illustrated by the case of a 38-year-old HIV-positive man with well-controlled infection. He developed disseminated *Salmonella enterica* infection, with bacteremia, septic arthritis, and ultimately corpus callosum involvement, following chronic self-administration of corticosteroids for cluster headaches. This case underscores that corticosteroid exposure can precipitate systemic dissemination even in patients with preserved CD4 counts. Although this condition carries a high risk of mortality, early recognition, targeted antibiotic therapy, and careful multidisciplinary management of underlying immunosuppression are critical to improving survival and minimizing neurological sequelae.

## 1. Introduction

*Salmonella* species (spp.) are facultative intracellular, Gram-negative bacilli and among the most common causes of foodborne illness worldwide [1,2]. In immunocompetent adults, infection is typically confined to the gastrointestinal tract, manifesting as acute, self-limiting gastroenteritis with diarrhea, fever, and abdominal pain. These cases generally resolve without antimicrobial therapy, and bacteremia is rare [3]. However, in vulnerable or immunocompromised hosts, *Salmonella* spp. can breach intestinal epithelial barriers, evade phagocytic clearance, and disseminate via the bloodstream to distant organs [4,5,6,7]. Disruption of this barrier is a hallmark and a risk factor for many intestinal and chronic inflammatory diseases, leading to microbial translocation and systemic inflammation, especially in the HIV population. A chronic inflammatory response persists in PWH even after long-term virological suppression during antiretroviral treatment. These dysfunctions ultimately facilitate the bloodstream dissemination of various infections, such as *Salmonella* spp. The resulting clinical syndromes, collectively termed invasive non-typhoidal *Salmonella* infections (iNTS), include bacteremia, osteomyelitis, septic arthritis, endocarditis, and, in exceedingly rare cases, central nervous system (CNS) involvement [2]. These infections are frequently associated with prolonged hospitalization, high healthcare costs, and significant morbidity.

CNS infections caused by *Salmonella* spp. are exceptionally rare in adults, accounting for only a tiny fraction of bacterial meningitis cases [8]. When they occur, these infections carry high mortality and often result in long-term neurological sequelae, including seizures, cognitive impairment, and focal deficits [3,4,5]. Their rarity, coupled with nonspecific early symptoms such as fever, malaise, or headache, frequently delays diagnosis, complicating clinical management and contributing to poor outcomes. Epidemiologically, CNS involvement is predominantly described in patients with predisposing immunosuppressive conditions, highlighting the importance of host immunity in disease susceptibility.

Historically, before the widespread adoption of combination antiretroviral therapy (ART), people living with HIV (PWH) were highly predisposed to invasive *Salmonella* spp. infections [8]. Recurrent *Salmonella* spp. septicemia was recognized as an AIDS-defining illness [9,10,11], with incidence rates among untreated PWH up to a thousand times higher than in the general population, particularly in resource-limited regions such as sub-Saharan Africa. ART has significantly reduced this burden by restoring immune function, improving mucosal integrity, and decreasing microbial translocation. Nevertheless, residual susceptibility persists, and even virologically suppressed individuals with preserved CD4+ counts may remain at risk if additional immunosuppressive conditions—such as malignancy, biologic therapies, or chronic corticosteroid use—are present [12].

Among these factors, corticosteroid exposure represents a particularly potent and often underrecognized contributor to invasive *Salmonella* spp. disease. Glucocorticoids exert broad immunosuppressive effects, impairing both innate and adaptive defenses. They inhibit macrophage activation, suppress neutrophil recruitment, reduce phagocytosis, and downregulate proinflammatory cytokine signaling. Consequently, the host’s ability to contain intracellular pathogens such as *Salmonella* spp. is compromised, facilitating bacterial persistence, systemic dissemination, and seeding of distant organs, including the CNS. In PWH, corticosteroid-induced immunosuppression is further amplified by HIV-associated dysregulation of cellular immunity, even when quantitative CD4+ counts are adequate. This dual impairment establishes a permissive environment for bacterial invasion of normally protected sites, increasing the risk of severe, life-threatening complications.

The pathogenesis of *Salmonella* spp. CNS infections are complex and multifactorial. Hematogenous dissemination appears to be a prerequisite for CNS seeding, with microbial invasion occurring via disrupted endothelial barriers or through infected macrophages diapedesis across the blood–brain barrier. Monocyte/macrophage transmigration across the Blood–Brain Barrier involves a tightly regulated, multi-step extravasation process (capture, rolling, arrest, crawling, diapedesis) driven by chemokines (like MCP-1/CCL2) and specific adhesion molecules (like CD31, CD99, CX3CR1), allowing these immune cells to enter the CNS during inflammation, where they differentiate into macrophages, clearing debris but potentially causing damage, with molecules like occludin on monocytes influencing this journey. Once within the CNS, *Salmonella* spp. can induce a range of pathological processes, from meningitis and ventriculitis to abscess formation and diffuse meningoencephalitis. The severity and extent of CNS involvement are influenced by pathogen virulence, host immune status, and the presence of additional comorbidities [4,5].

This systematic review integrates a detailed clinical case of disseminated *Salmonella enterica* infection with CNS involvement in a HIV-positive adult chronically exposed to corticosteroids, alongside a synthesis of published literature on similar cases. By combining case-based insights with evidence from broader epidemiological and clinical studies, we aim to provide a comprehensive understanding of the mechanisms underlying *Salmonella* spp. neuroinvasion in immunocompromised adults, highlight diagnostic and therapeutic challenges, and outline strategies to optimize clinical outcomes. This dual perspective underscores the importance of recognizing overlapping immunosuppressive factors—particularly corticosteroid use and HIV-infection—as key determinants of disease severity and dissemination [13]. Furthermore, it emphasizes the necessity for heightened clinical vigilance, early neuroimaging, and prompt initiation of targeted antimicrobial therapy in high-risk populations to mitigate morbidity and mortality.

## 2. Methods

A systematic review of the literature was conducted in accordance with the Preferred Reporting Items for Systematic Reviews and Meta-Analyses (PRISMA) 2020 guidelines. The primary aim of the review was to identify and synthesize all reported cases of CNS infections caused by *Salmonella* spp. in immunocompromised adults, with particular emphasis on cases associated with HIV infection, in order to provide a comprehensive understanding of clinical presentation, risk factors, and outcomes.

A comprehensive search strategy was applied to three major electronic databases: PubMed/MEDLINE, Embase, and the Cochrane Library, covering all publications from database inception to October 2025. Both controlled vocabulary (MeSH and Emtree terms) and free-text keywords were used, including “*Salmonella*,” “HIV,” “central nervous system,” “meningitis,” and “immunosuppression.” Boolean operators and truncations were applied to maximize sensitivity while maintaining specificity, and the search strategy was tailored to each database to ensure optimal coverage. GT and MB independently screened all titles and abstracts retrieved in the initial search, followed by full-text assessment of potentially eligible studies. Discrepancies were resolved by consensus or consultation with a third investigator. Additionally, reference lists of included articles were manually reviewed to identify relevant studies not captured electronically, enhancing the completeness of case identification.

Inclusion criteria were predefined. Eligible studies comprised case reports, small case series, and observational studies describing adult patients (≥18 years) with laboratory-confirmed CNS infection due to *Salmonella* spp., defined as isolation of the microorganism from cerebrospinal fluid (CSF), brain abscess aspirate, or postmortem CNS tissue. Only cases occurring in the context of documented immunosuppression were included. Recognized immunosuppressive conditions encompassed HIV-infection (regardless of CD4+ count or viral load), chronic corticosteroid therapy, chemotherapy, malignancy, organ transplantation, or treatment with biologic or targeted immunomodulatory agents. Exclusion criteria included pediatric cases (<18 years), animal studies, articles in languages other than English, reviews without original data, and reports of *Salmonella* spp. infections limited to non-CNS sites (e.g., bacteremia, osteomyelitis, or endocarditis) without neurological involvement.

Data extraction was performed independently by the same authors using a standardized collection template specifically designed for this review. Extracted variables included patient demographics (age, sex, geographic origin, year of publication); underlying immunosuppressive conditions; the identified *Salmonella* spp., when available; and source of microbiological confirmation (CSF, brain abscess, blood, or stool). Clinical variables included neurological symptoms, extra-CNS manifestations, and duration of illness before diagnosis. Imaging findings were summarized from CT and MRI reports, while treatment data encompassed antimicrobial regimens, duration of therapy, and any surgical interventions. Clinical outcomes, including survival, neurological sequelae, and relapse, were systematically recorded. Quantitative data were summarized descriptively, and qualitative synthesis was used to identify recurring patterns in clinical presentation, disease progression, and management. Cases reported prior to the introduction of combination antiretroviral therapy were analyzed separately to contextualize historical trends in HIV-associated *Salmonella* spp. CNS infections.

**Figure 1:**
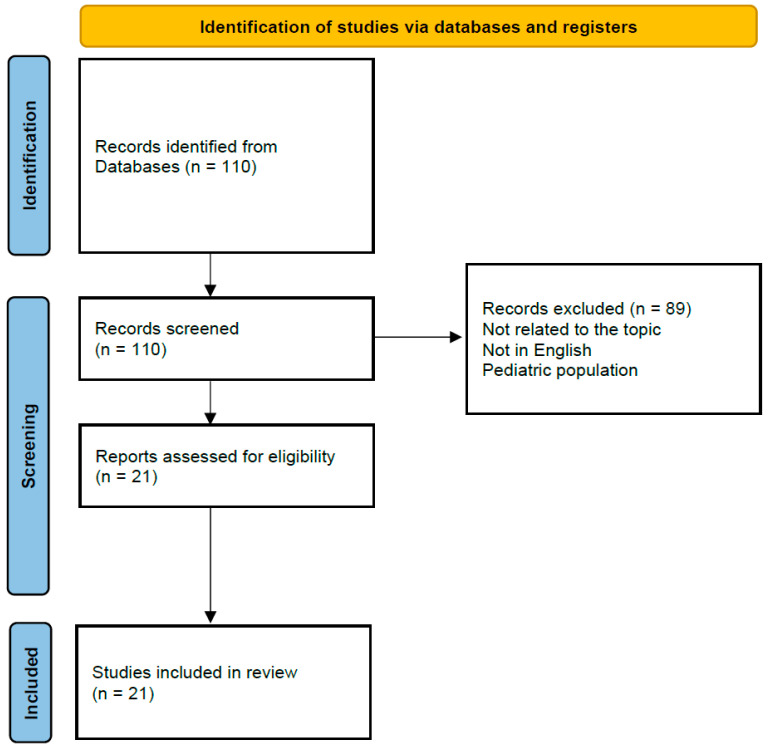
Prisma flow diagram.

The review process adhered to the PRISMA framework: after duplicate removal, records were screened at the title/abstract level, full-text eligibility was assessed, and reasons for exclusion were documented. A PRISMA flow diagram was generated to illustrate the study selection process (Figure 1). Using this systematic approach, 18 adult cases meeting the inclusion criteria were identified across 27 publications from 1972 to 2025. The synthesis provides a comprehensive overview of the epidemiologic context, diagnostic challenges, therapeutic strategies, and prognostic outcomes of *Salmonella* spp. CNS infections in immunocompromised adults, emphasizing the importance of early recognition, targeted antimicrobial therapy, and careful management of underlying immunosuppressive conditions to improve survival and reduce neurological sequelae.

## 3. A Patognomonic Clinical Case

In August 2024, a 38-year-old man living with HIV presented to the Emergency Department with a 10-day history of profuse watery diarrhea exceeding fifteen stools per day, accompanied by severe fatigue, diffuse myalgia, and an unintentional weight loss of 7 kg. He reported no recent antibiotic use and denied traveling outside of Southern Italy. He did not report any exposure to possible contaminated food, such as unpasteurized products, eggs, or poultry. His HIV infection was diagnosed in August 2022. He was on a single-tablet regimen of bictegravir, emtricitabine, and tenofovir alafenamide, with excellent adherence, durable virologic suppression, and a stable CD4+ count consistently above 1200 cells/mm^3^.

The patient had a long-standing history of chronic migraine and probable cluster headache. In the preceding three months before admission, he had self-administered repeated courses of high-dose oral prednisone (25–50 mg daily for several weeks at a time) without medical supervision. This chronic corticosteroid exposure resulted in iatrogenic Cushing’s syndrome, clinically manifested as central obesity, moon facies, dorsocervical fat pad, and violaceous striae. Biochemical evaluation at that point revealed mild hypercholesterolemia (total cholesterol 5.5 mmol/L, normal level < 5 mmol/L) and impaired fasting glucose (6.4 mmol/L, normal level 4.1–6.1 mmol/L). Other blood tests were in the normal range (CD4+: 1214 cells/mmc, 29%; HIV-RNA < 20 cp/mL). No prophylactic antimicrobial measures or endocrinologic follow-up were implemented. The prolonged steroid use likely contributed to functional impairment of both innate and adaptive immunity, increasing susceptibility to pathogens. Specifically, glucocorticoids inhibit macrophage activation, phagocytosis, and cytokine-mediated immune responses, while simultaneously attenuating neutrophil trafficking, creating a permissive environment for bacterial persistence and dissemination.

Upon admission, laboratory assessment revealed acute kidney injury (creatinine 2.2 mg/dL), hypokalemia (3.1 mmol/L), hyponatremia (129 mmol/L), and markedly elevated inflammatory markers, including C-reactive protein at 18.4 mg/dL (normal < 5 mg/dL). Blood and stool cultures grew non-typhoidal *Salmonella enterica*, confirming disseminated infection with enteric involvement. The patient was initiated on intravenous rehydration and ceftriaxone (2 g daily). The isolate was resistant to ciprofloxacin but fully susceptible to other antimicrobials, with favorable minimum inhibitory concentrations (ceftriaxone 0.064 mg/L; meropenem 0.016 mg/L; cotrimoxazole 0.019 mg/L). Corticosteroid treatment was tapered after hospitalization and completely discontinued after 3 weeks. This therapy produced partial improvement: diarrhea frequency decreased, and inflammatory markers transiently declined. However, persistent fever and malaise suggested incomplete microbial clearance. This pattern is consistent with prior literature, which demonstrates that immunocompromised hosts, particularly those receiving corticosteroids, are prone to prolonged bacteremia and relapsing infection due to impaired intracellular killing and persistence of *Salmonella* spp. within the reticuloendothelial system [14,15].

During the second week of hospitalization, the patient developed painful swelling of both wrists and ankles. Musculoskeletal ultrasound of the left shoulder revealed synovial effusion with marked cutaneous hyperemia, and arthroscopic aspiration confirmed purulent arthritis. Synovial fluid culture again yielded *Salmonella enterica*, establishing the diagnosis of septic arthritis. Musculoskeletal involvement occurs in approximately one-third of adult iNTS cases, particularly in patients with impaired cellular immunity or preexisting joint abnormalities. Despite ongoing ceftriaxone therapy, intermittent fevers recurred, accompanied by right knee effusion and elevated inflammatory markers, necessitating three additional arthroscopic interventions. This stepwise progression underscores the pathogenic potential of *Salmonella* spp. to disseminate via the bloodstream and seed multiple organ systems in immunocompromised hosts.

Twenty days after symptom onset, the patient developed new neurological symptoms, including progressive confusion, somnolence, and disorientation, raising concern for CNS involvement. Neurological examination revealed mild neck stiffness but no focal deficits. Lumbar puncture yielded turbid CSF with neutrophilic pleocytosis (850 cells/μL), markedly elevated protein (1220 mg/dL), and hypoglycorrhachia (glucose 28 mg/dL). Molecular testing for viral pathogens (Biofire FilmArray and PCR for HSV-1, HSV-2, VZV) was negative. After two days of incubation, the culture of CSF grew *Salmonella enterica*, confirming bacterial meningitis.

**Figure 2:**
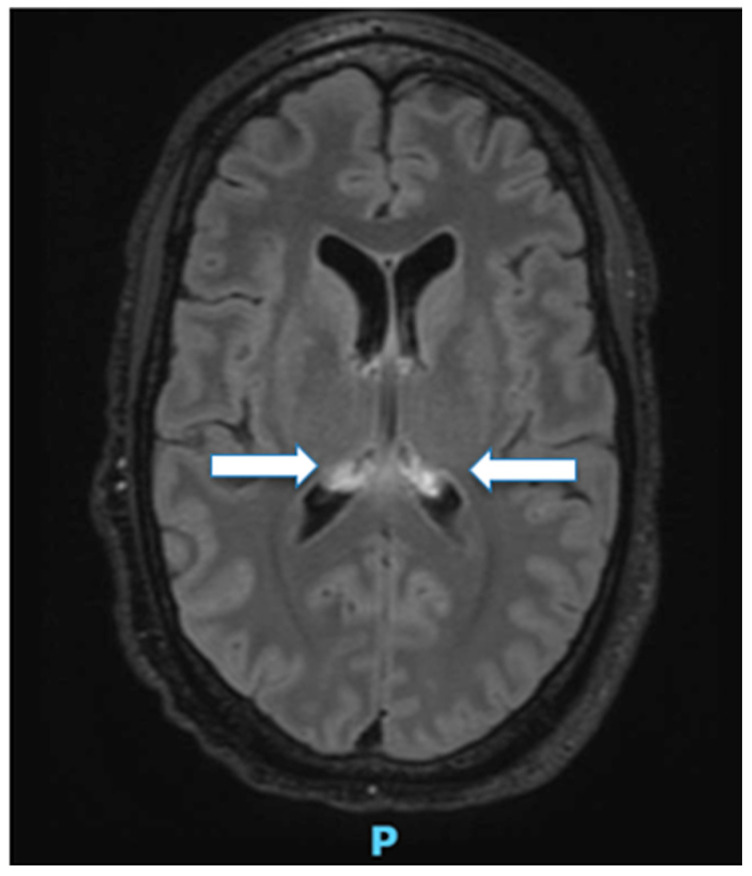
CLOCCS of the corpus callosum.

Magnetic resonance imaging (MRI) of the brain revealed bilateral hyperintensities in the corpus callosum and splenium, consistent with cytotoxic lesions of the corpus callosum (CLOCCs) (Figure 2). CLOCCs are typically reversible lesions secondary to metabolic stress or inflammatory processes and are most often seen in the splenium. They have been described in various contexts, including viral infections (e.g., influenza, Epstein–Barr virus, COVID-19), metabolic derangements (hypoglycemia), exposure to chemotherapeutic agents or toxins (e.g., 5-fluorouracil), seizures, antiepileptic drug withdrawal, cerebrospinal fluid disturbances, and rarely bacterial infections [16,17,18,19]. The pathophysiology is thought to involve a cascade of proinflammatory cytokines (IL-1, IL-6, TNF-α) and glutamate-mediated excitotoxicity, leading to cytotoxic edema of myelinated fibers, particularly in regions rich in glutamate receptors such as the splenium. In the context of *Salmonella* CNS infection, these lesions likely reflect transient inflammatory and metabolic stress rather than direct bacterial invasion [16].

Given the patient’s deterioration and the multi-organ involvement despite ceftriaxone therapy, antibiotic treatment was escalated to high-dose intravenous meropenem (2 g every 8 h) for six weeks. Supportive care included meticulous electrolyte management, aggressive hydration, and close monitoring for potential secondary complications. Over the course of therapy, the patient gradually improved, achieving defervescence within ten days and complete neurological recovery by hospital discharge. A lumbar puncture repeated at 2 and 4 weeks showed improvement in all parameters, with normalization of glucose and protein levels and no further leukocytosis. At 12-month follow-up, he remained fully recovered, with no residual neurological or musculoskeletal deficits.

This case highlights the complex interplay between well-controlled HIV infection and exogenous corticosteroid-induced immunosuppression in facilitating invasive *Salmonella* spp. disease. Although antiretroviral therapy dramatically reduces the risk of iNTS bacteremia among PWH, secondary immunosuppressive factors, particularly chronic steroid exposure, can restore susceptibility to severe disseminated infections. The clinical course, progressing from enteric infection to bacteremia, septic arthritis, and meningitis with CLOCCs, mirrors the stepwise dissemination pattern described in case-based literature and underscores the need for vigilance in patients with overlapping immunosuppressive risk factors.

Several key lessons emerge from this case. First, clinicians should recognize that even PWH with high CD4+ counts and viral suppression might develop life-threatening infections if additional immunosuppressive factors are present. Second, aggressive antimicrobial therapy with agents achieving adequate CNS penetration is critical for favorable outcomes. Third, a multidisciplinary approach—including infectious disease, neurology, rheumatology, orthopaedics, and endocrinology input—is essential to address both the infection and the underlying immunosuppressive state. Finally, early recognition of secondary immunosuppression due to chronic corticosteroid use is crucial to prevent dissemination and reduce morbidity and mortality associated with iNTS infections.

## 4. Systematic Review of the Literature

A total of 21 papers met the inclusion criteria, comprising twelve single-case reports, three small case series, and three retrospective cohort studies, for an overall cohort of thirty-three adult patients with confirmed *Salmonella* spp. CNS infections in the context of immunosuppression (Table 1). The temporal distribution of reports spanned from 1972 to 2025, with a noticeable increase in documented cases over the past two decades, likely reflecting improved diagnostic capabilities and heightened awareness of atypical *Salmonella* spp. presentations in immunocompromised hosts [20,21,22,23,24,25,26,27,28,29,30,31,32,33,34,35,36,37,38,39,40].

Immunosuppressive conditions were heterogeneous. HIV-infection accounted for fourteen cases (46.7%), representing the largest subgroup [14,15,16,17,18,19,20,21,22,23,24]. Seven cases (23.3%) occurred in patients receiving biologic or targeted immunomodulatory therapy, most commonly tumor necrosis factor-alpha inhibitors for autoimmune diseases such as rheumatoid arthritis or Crohn’s disease [37,38,39]. Chronic corticosteroid exposure was reported in eight patients (24%), while six patients (18%) had underlying malignancy or were receiving cytotoxic chemotherapy. Several patients exhibited overlapping risk factors, such as concurrent HIV-infection and corticosteroid therapy, highlighting the multifactorial nature of host susceptibility. Patient ages ranged from 19 to 79 years, with a median of 46 years, and there was a slight male predominance (61%).

Clinically, most patients presented with meningitis-like symptoms. Headache, fever, and nuchal rigidity were reported in approximately 70% of cases, while 40% experienced altered mental status, confusion, or seizures [26,40]. A smaller proportion developed focal neurological deficits related to abscess formation or ventriculitis. In several reports, initial gastrointestinal symptoms preceded neurological manifestations by one to two weeks, reinforcing the hypothesis that CNS involvement represents a late complication of disseminated infection [40]. In case of severe systemic infections, *Salmonella* spp. can be found in blood/CSF, with studies showing high detection rates in CSF (~50%) and blood (~72%) in invasive cases, often alongside fecal carriage, highlighting the need for diverse specimen cultures (blood, stool, CSF, bone marrow, bile) for accurate diagnosis.

Radiological findings varied considerably. Brain MRI and CT frequently demonstrated ventriculitis, cerebral abscesses, or subdural empyema. In a subset of patients, cytotoxic lesions of the corpus callosum (CLOCCs) were observed, representing radiological markers of inflammatory or metabolic stress within myelinated fibers [20,31]. Although nonspecific, these lesions have been increasingly associated with bacterial meningitis and may indicate reversible inflammatory injury in *Salmonella* spp. neuroinfection.

CSF analysis uniformly demonstrated features of bacterial meningitis, characterized by neutrophilic pleocytosis, markedly elevated protein, and hypoglycorrhachia. Despite these findings, *Salmonella* spp. isolation from CSF was inconsistent—positive in roughly half of the reported cases. In contrast, blood cultures were positive in 72%, supporting the hypothesis that hematogenous dissemination and persistent bacteremia precede CNS invasion [4].

Microbiological data identified *Salmonella enterica* serovars *enteritidis* and *typhimurium* as the predominant pathogens, together accounting for over 80% of isolates. Sporadic cases involved *S. dublin*, *S. choleraesuis*, and *S. heidelberg*. Antimicrobial susceptibility testing revealed fluoroquinolone resistance in 22% of isolates, highlighting emerging resistance patterns and the enduring therapeutic relevance of third-generation cephalosporins such as ceftriaxone or cefotaxime [4,41,42,43]. In Switzerland, in 2025, the prevalence of *Salmonella* spp. strains resistant to fluoroquinolones was 18%, whereas resistance to ceftriaxone or carbapenems remained < 1% [44].

Treatment regimens varied across studies, reflecting temporal differences and individual case complexity. Ceftriaxone was the most frequently administered agent, often combined with a fluoroquinolone or aminoglycoside for a synergistic effect. Duration of antimicrobial therapy ranged from four to twelve weeks, with longer courses favored in cases involving abscesses or ventriculitis. Surgical drainage or debridement was required in 24% of patients, particularly those with localized collections or obstructive hydrocephalus [40].

Outcomes remained poor despite aggressive management, with an overall mortality rate of 33%. Among survivors, 39% experienced persistent neurological sequelae, including focal weakness, cognitive deficits, or chronic seizure disorders. Favorable outcomes were more likely in patients receiving early diagnosis, prompt initiation of combination antibiotic therapy, and effective control of the underlying immunosuppressive condition. Collectively, these findings emphasize that *Salmonella* spp. CNS infections, though rare, represent a severe and life-threatening clinical condition, particularly in immunocompromised adults.

## 5. Discussion

Our case, integrated with a systematic review of the literature, reveals converging patterns in the pathogenesis, clinical course, and management of CNS infections caused by *Salmonella* spp. in immunocompromised adults. Although exceptionally rare, CNS involvement almost invariably develops in the context of systemic disease, often preceded by bacteremia and gastrointestinal localization [4].

Immunosuppression emerges as the principal predisposing factor. In our review, advanced HIV-infection was the most frequently reported underlying cause of immunosuppression in patients developing CNS complications due to *Salmonella* spp. Importantly, even patients with virologically suppressed HIV and preserved CD4+ counts remain at risk for severe disease when additional immunosuppressive insults coexist. Chronic corticosteroid exposure, in particular, synergistically compromises host defenses. In the cases analyzed, corticosteroid therapy frequently preceded disseminated infection, delayed bacterial clearance, and was associated with relapse, highlighting its critical role in the pathophysiology of severe iNTS disease. Biologic therapies and cytotoxic chemotherapy also contributed to susceptibility; however, the combination of HIV-related immune dysfunction and corticosteroid-induced immunosuppression represented a particularly high-risk pattern.

Mechanistically, the interplay of HIV-associated immune dysregulation and glucocorticoid-mediated suppression generates multiple vulnerabilities. Corticosteroids blunt both innate and adaptive immunity by impairing macrophage activation, reducing phagocytosis, inhibiting neutrophil chemotaxis, and downregulating proinflammatory cytokine production. HIV infection further compromises T-cell–mediated immunity and disrupts mucosal barrier integrity, facilitating pathogen translocation. Together, these factors permit intracellular persistence of *Salmonella* spp. within macrophages, prolonged bacteremia, and eventual seeding of distant organs, including the CNS. Microvascular injury and endothelial dysfunction within the meninges facilitate bacterial entry into neural tissue, where *Salmonella* spp. triggers intense neutrophilic inflammation, endothelial injury, and microabscess formation. Neuroimaging frequently reflects this cascade: cytotoxic lesions of the corpus callosum (CLOCCs), ring-enhancing abscesses, and ventriculitis indicate focal edema and cellular injury secondary to localized cytokine-mediated toxicity [16].

Clinically, these pathophysiologic mechanisms manifest as a spectrum of overlapping systemic and neurological features. Across our case and the reviewed literature, fever often persists or recurs despite appropriate antimicrobial therapy, reflecting ongoing bacteremia or delayed immunologic clearance [45,46]. Neurological symptoms—including confusion, lethargy, seizures, and focal deficits—may appear subtly or late in the course of illness. Musculoskeletal involvement, such as reactive or septic arthritis, frequently accompanies CNS disease and may serve as an early clinical warning of disseminated infection. Recognizing these patterns is crucial: in immunocompromised adults with *Salmonella* bacteremia, persistent or relapsing fever, particularly when paired with musculoskeletal or subtle neurological signs, should prompt early neuroimaging and lumbar puncture even in the absence of overt neurological deficits.

Third-generation cephalosporins remain the cornerstone of antibiotic treatment, with ceftriaxone being the most commonly used agent. Given the pathogen’s intracellular persistence and risk of CNS relapse, prolonged therapy—often exceeding six weeks—is recommended. Carbapenems, particularly meropenem, are suggested for resistant or relapsing cases due to their broad-spectrum coverage and CNS penetration. In our case, we elected to switch to meropenem rather than increase the dose of ceftriaxone, despite its adequate susceptibility, due to the multi-organ dissemination of the infection. Surgical intervention, including drainage of abscesses or empyemas, is essential in select patients to optimize bacterial clearance. Equally critical is the management of underlying immunosuppressive factors. Tapering or discontinuing corticosteroids when feasible can restore immune competence and reduce recurrence risk. This approach underscores the necessity of multidisciplinary care involving infectious disease specialists, neurologists, rheumatologists, and endocrinologists to address the complex interplay of systemic infection, CNS involvement, and host immunity.

Despite advances in antimicrobial therapy and supportive care, prognosis remains poor. Literature data indicate that approximately one-third of patients succumb to *Salmonella* spp. CNS infection, reflecting the challenges of timely diagnosis and the severity of systemic dissemination. Among survivors, over one-third experience persistent neurological sequelae, including cognitive impairment, focal motor deficits, and seizures. These findings highlight the high morbidity associated with CNS involvement and the critical importance of prevention, early recognition, and vigilant monitoring, particularly in PWH. Careful assessment of immunosuppressive medication use, prompt recognition of gastrointestinal or systemic *Salmonella* spp. infection, and early escalation of therapy are key strategies to prevent CNS dissemination and improve outcomes.

Our clinical case mirrors these observations. The patient’s progression from profuse diarrhea and bacteremia to musculoskeletal and CNS involvement exemplifies the stepwise dissemination typical of iNTS infection in immunocompromised hosts. MRI findings of CLOCCs correlated with cytotoxic inflammatory injury described in other cases. Prolonged intravenous antimicrobial therapy combined with careful tapering of corticosteroids facilitated complete recovery without residual deficits. This case reinforces that even PWH with preserved immune function remain susceptible to severe invasive *Salmonella* spp. infections when additional immunosuppressive factors are present, highlighting a critical area for clinical vigilance.

In summary, *Salmonella* spp. CNS infections in immunocompromised adults represent a rare but devastating complication of systemic disease. The convergence of HIV infection and corticosteroid exposure markedly elevates risk, and CNS involvement is typically preceded by bacteremia and may be heralded by musculoskeletal or systemic inflammatory signs. Clinicians should maintain a high index of suspicion for neurological complications in any immunocompromised adult with persistent or relapsing bacteremia. Early neuroimaging, lumbar puncture, prolonged targeted antimicrobial therapy, and judicious management of immunosuppressive medications are central to optimizing outcomes and minimizing long-term morbidity. Integration of clinical cases with systematic literature review underscores the dynamic interplay between host immunity, pathogen virulence, and therapeutic intervention, providing a framework for early recognition, effective treatment, and improved prognosis in this life-threatening condition.

## 6. Conclusions

CNS infections caused by *Salmonella* spp. remain an exceptionally rare but highly severe manifestation of (iNTS) disease in immunocompromised adults. The integration of our clinical case with the existing literature underscores a critical and often underappreciated insight: even PWH who maintain preserved CD4+ counts and durable virologic suppression remain vulnerable to life-threatening dissemination when additional immunosuppressive factors, particularly chronic corticosteroid exposure, are present. This observation highlights the delicate balance between host immunity and pathogen virulence, in which subtle perturbations of immune homeostasis can precipitate severe systemic involvement and CNS seeding.

Early recognition of CNS complications is paramount. Persistent or relapsing fever, new-onset neurological symptoms, or musculoskeletal manifestations in the context of *Salmonella* spp. bacteremia should trigger immediate neuroimaging and cerebrospinal fluid analysis. Magnetic resonance imaging, particularly the detection of CLOCCs, offers crucial diagnostic and prognostic information, reflecting inflammatory or metabolic stress within myelinated fibers rather than direct bacterial invasion. Timely initiation of targeted, prolonged antimicrobial therapy—primarily third-generation cephalosporins, with escalation to carbapenems in resistant or relapsing cases—is essential to control infection and prevent relapse. In selected cases, surgical intervention may be required to manage abscesses, empyemas, or obstructive hydrocephalus.

Equally important is the careful management of immunosuppressive exposure. Gradual tapering or temporary discontinuation of corticosteroids, along with optimization of underlying conditions such as HIV infection, can significantly reduce the risk of persistent or recurrent disease. A multidisciplinary approach—integrating infectious disease, neurology, rheumatology, and endocrinology—is critical to ensure comprehensive care and to address both the systemic infection and the patient’s immunologic status.

Beyond individual patient management, these findings have broader clinical and public health implications. Heightened awareness of the risk posed by overlapping immunosuppressive factors, judicious corticosteroid use, and early initiation of appropriate antimicrobial therapy are central to reducing morbidity and mortality from invasive *Salmonella* disease. Vigilant monitoring of high-risk populations, combined with timely intervention, can improve outcomes even in rare but severe presentations such as CNS involvement.

In conclusion, CNS *Salmonella* spp. infections in immunocompromised adults constitute a high-stakes clinical scenario. Prevention and management require early recognition of systemic and neurological signs, timely, appropriately prolonged antimicrobial therapy, and careful modulation of immunosuppressive factors. This case, alongside the literature, reinforces a critical paradigm in contemporary medicine: successful outcomes rely not only on antimicrobial strategies but also on comprehensive evaluation of host susceptibility and the implementation of coordinated, multidisciplinary care. Recognition of these principles is essential to mitigate the substantial morbidity and mortality associated with this life-threatening condition.

## Figures and Tables

**Table 1:** Data extraction summary of included studies on *Salmonella* CNS infections in immunocompromised adults. Abbreviations: CSF: Cerebrospinal fluid; CT: Computed Tomography; MRI: Magnetic Resonance; NR: Not reported; CNS: Central Nervous System; ART: Antiretroviral Therapy; GCS: Glasgow Coma Scale; ICU: Intensive Care Unit.

Study	Study Design/Setting/Year	N Patients (CNS)	Cause of Immunosuppression	HIV Status	Symptoms/Clinical Presentation	Diagnostic Method (CSF/Culture/Imaging)	Outcome
Ahmed et al. [20]	Case report	1	HIV infection	CD4+: 10 cells/mmc HIV-RNA: NR	Confusion, ventriculitis, brain abscess(es)	Blood and CSF culture positive; CT/MRI	Gradual improvement; discharged after prolonged admission
Leonard et al. [21]	Case report + literature review	1	HIV infection	CD4+: 294 cells/mmc HIV-RNA: 24,666 cp/mL	Fever, headache, neck stiffness	CSF culture positive (*Salmonella infantis*)	Recovery; no relapse at 12 months
Xu et al. [22]	Retrospective case series	NR (CNS subset not explicit in abstract)	HIV infection (CD4 < 100 associated)	CD4+: 20 cells/mmc (median) HIV-RNA: <100 IU/mL: 10	Nonspecific invasive *Salmonella* presentations	Blood/CSF culture; serotyping	Variable; antimicrobial resistance concern
Keddy et al. [23]	Surveillance/case series	278 iNTS meningitis cases identified	High HIV prevalence among adult cases	CD4+ < 200 cells/mmc: 26 HIV-RNA: NR	Typical meningitis; low GCS predicts mortality	CSF/blood cultures; serotyping	Mortality associated with clinical severity
Belloso et al. [24]	Case report + mini-review	1 (recurrent meningitis with subarachnoid hemorrhage)	HIV infection)	CD4+: 341 cells/mmc HIV-RNA: 3.7–4.2 log cp/mL	Recurrent meningitis (multiple episodes)	CSF cultures and imaging across episodes	Variable; highlights recurrence risk
Subramoney [25]	Review/Forum	NR (review)	HIV infection (discusses iNTS in HIV-positive adults)	CD4+: 96 cells/mmc HIV-RNA: suppressed	Bacteraemia and focal complications, including meningitis (rare)	Culture-based diagnosis	Review-level statements (higher complication rates in HIV)
Fernández-Guerrero et al. [26]	Case series + review	10 focal infections among HIV patients (includes meningitis/abscess)	HIV/AIDS (many with CD4 < 100)	CD4+: 172 cells/mmc (mean) HIV-RNA: NR	Focal suppurative infections, including intracranial disease	Culture from focus/CSF; imaging for abscesses	High morbidity/mortality (pre-ART era)
Hajra et al. [27]	Narrative review on iNTS strains	NR (microbiologic focus)	Discusses host factors, including HIV	CD4+: NR HIV-RNA: NR	Extra-intestinal invasive disease is described	Molecular/serotyping methods discussed	NR
Katsenos et al. [28]	Case report	1	Newly diagnosed HIV infection during workup	CD4+: 16 cells/mmc HIV-RNA: NR	Fever, nuchal rigidity, confusion; progressed to septic shock	CSF and blood cultures were positive for *S. enteritidis*	Severe course with septic shock; details in full text
Gutiérrez et al. [29]	Case report	1 (recurrent meningitis in an AIDS patient)	AIDS	CD4+: NR HIV-RNA: NR	Recurrent meningitis despite therapy	CSF culture positive	Recurrence
Elton et al. [30]	Case report	1	New AIDS diagnosis	CD4+: 3 cells/mmc HIV-RNA: 560,000 cp/mL	Altered mental status, fever; cloudy CSF with Gram-negative rods	CSF culture positive for *S. enterica*	ICU care required; case report documents clinical course
Sait et al. [31]	Case report	1 (intracranial abscess post-glioblastoma resection)	Post-surgical (iatrogenic), not HIV-related	HIV negative	Post-op focal deficits; imaging consistent with abscess	Imaging and abscess culture positive for *S. enteritidis*	Recovery after intervention
Swe et al. [32]	Case report	1	AIDS	CD4+: 2 cells/mmc HIV-RNA: 99,658 cp/mL	Fever, diarrhea, neck stiffness, oral thrush	CSF and blood culture were positive for *S. typhimurium*	NR
Grant et al. [33]	Hospital-based study	NR (epidemiologic study of HIV opportunistic disease)	HIV infection	CD4+: NR HIV-RNA: NR	Opportunistic infections and spectrum reported	NR	NR
Mbelesso et al. [34]	Retrospective study	502 adult meningitis cases over 5 years (not *Salmonella*-specific)	HIV discussed regionally; not *Salmonella*-specific	CD4+: NR HIV-RNA: NR	Typical bacterial meningitis presentations; seasonal peaks	Hospital diagnostics; culture data summarized	NR
Zouiten et al. [35]	Retrospective cohort	92 women with AIDS in the cohort; CNS *Salmonella* cases not explicitly listed	AIDS	CD4+: NR HIV-RNA: NR	Spectrum of opportunistic infections; high proportion of AIDS	NR	NR
Cohen [36]	Review;	NR	HIV/AIDS	CD4+: NR HIV-RNA: NR	Imaging patterns described for intracranial bacterial disease	Neuroimaging + culture correlation	NR
Dower et al. [37]	Case report	1 (elderly female)	TNF-antagonist therapy for rheumatoid arthritis (iatrogenic)	HIV negative	Back/leg pain, then meningitis signs; altered function	CSF and blood cultures were positive for *Salmonella* spp.	Slow improvement; illustrates non-HIV immunosuppression risk
Rezaei et al. [38]	Scoping review;	NR	Immunocompromised patients	CD4+: NR HIV-RNA: NR	Varied; reports included *Salmonella* species in case reports/series	Depends on included reports	NR
Ikejiri et al. [39]	Case report	1	Influenza A immediately prior to infection	CD4+: NR HIV-RNA: NR	Sepsis, meningitis, vertebral osteomyelitis	Blood/CSF cultures; imaging for osteomyelitis	Recovery
Gille-Johnson et al. [40]	Case report	1	None reported	CD4+: NR HIV-RNA: NR	Meningitis features	CSF culture positive for *S. virchow*	Full recovery

## Data Availability

Not applicable.
